# Contracting with private providers for primary care services: evidence from urban China

**DOI:** 10.1186/2191-1991-3-1

**Published:** 2013-01-17

**Authors:** Yan Wang, Karen Eggleston, Zhenjie Yu, Qiong Zhang

**Affiliations:** 1 Shandong Provincial Health Department, Division of Disease Control, 9 Yang Dong Xin Lu, Shandong, 250014, China; 2 Asia Health Policy Program, Walter H. Shorenstein Asia-Pacific Research Center, Stanford University, 616 Serra St., Encina Hall E311, Stanford, CA, 94305-6055, USA; 3 Weifang Medical University, Weifang, Shandong, China; 4 Central University of Finance and Economics, School of Economics, Beijing, China

**Keywords:** Private providers, Contracting, Ownership, Primary care, Prevention, China

## Abstract

Controversy surrounds the role of the private sector in health service delivery, including primary care and population health services. China’s recent health reforms call for non-discrimination against private providers and emphasize strengthening primary care, but formal contracting-out initiatives remain few, and the associated empirical evidence is very limited. This paper presents a case study of contracting with private providers for urban primary and preventive health services in Shandong Province, China. The case study draws on three primary sources of data: administrative records; a household survey of over 1600 community residents in Weifang and City Y; and a provider survey of over 1000 staff at community health stations (CHS) in both Weifang and City Y. We supplement the quantitative data with one-on-one, in-depth interviews with key informants, including local officials in charge of public health and government finance.

We find significant differences in patient mix: Residents in the communities served by private community health stations are of lower socioeconomic status (more likely to be uninsured and to report poor health), compared to residents in communities served by a government-owned CHS. Analysis of a household survey of 1013 residents shows that they are more willing to do a routine health exam at their neighborhood CHS if they are of low socioeconomic status (as measured either by education or income). Government and private community health stations in Weifang did not statistically differ in their performance on contracted dimensions, after controlling for size and other CHS characteristics. In contrast, the comparison City Y had lower performance and a large gap between public and private providers. We discuss why these patterns arose and what policymakers and residents considered to be the main issues and concerns regarding primary care services.

## Background

### Introduction

Controversy surrounds the role of the private sector in delivering basic health services covered by public financing, especially in developing and transitional economies. The economic theory of contracting out [1] suggests that although private providers generally have stronger incentives for cost control, whether their quality is higher or lower than that of public providers depends on organizational incentives. Concerns about the uneven quality of private providers are frequent among policymakers [2]. Loevinsohn and Harding [3] argue that contracting for health service delivery from the private sector can improve quality and access, and a recent systematic review does find a positive impact of private providers on the quality of primary or public health services for the poor [4]. Yet it is not always clear how sensitive these results are to the institutional context of contracting, and the overall evidence base remains relatively thin.

In urban China, private providers play an important minority role in supplying primary health care services. The national health reforms begun in 2009 emphasize strengthening primary care and call for non-discrimination against private providers [5]. Statements by top Chinese officials about the next phase of health reforms beginning in 2012 also emphasize the importance of encouraging private participation in health service delivery, as well as continuing to strengthen primary care and link payment to measures of accountability and performance. However, formal contracting initiatives that incorporate private providers remain few, and the associated empirical evidence is very limited [6-10].

To help lay the foundation for filling this evidence gap, the present study provides some descriptive information about public and private sector involvement in primary care in urban China. We collect data on patients and providers affected by a natural experiment in contracting with private providers for urban primary and preventive health services in Shandong Province, China. In June 2008, the city of Weifang used a public bidding process to select and contract with 63 community health stations (CHS) – 30 government-owned and 33 private facilities. The contract stipulated payment from public funds for designated services, inclusion in the expanded social insurance coverage system, and the ability to receive professional training and financial assistance for investments in infrastructure. All buildings and medical equipment purchased through the program revert to the government at the end of the contract period. In exchange, contracted providers must adhere to policies governing service provision, quality of care, and regulatory oversight.

Our study uses routine monitoring data collected by the Weifang and provincial departments of health, supplemented by additional survey data from a subsample of patients and providers, to describe the implementation process of this contracting-out experiment and to outline a research agenda for future studies that aim to estimate the impact of contracting policies. The paper describes trends in data spanning one year before and after the policy implementation in Weifang and a neighboring city of similar size that did not contract with private providers on equal terms with government providers. Our case study addresses the following questions: What motivated local officials to contract with public and private providers? What measures of performance were included in the contract? Were there systematic differences in the patient populations served by providers of different ownership forms? What were the trends in the measured dimensions of performance? How did policymakers, providers, and patients perceive the quality of primary and preventive services at providers of different ownership? And what additional information should be gathered at baseline, during and after implementation if local governments and independent scholars wish to determine the causal impact of such contracting initiatives? This case study from urban Shandong may help to inform future analyses of China’s policies and those of other low- and middle-income countries seeking to integrate private providers into systems for better access and quality at affordable cost.

The paper is organized as follows. The next section provides background information on China’s health system, the limited role of private sector delivery, and recent policies supporting primary health care. We then describe the 2008 contracting reform in Weifang, our quantitative and qualitative data about that reform, and our methods for analyzing the data to control for confounding factors. The final sections present our descriptive results about the Weifang case study and discuss policy implications.

#### China’s health reforms and the role of private health service providers

China’s economic reforms have spurred unprecedented economic growth and improved living standards, but the transition to a market-oriented economy has also been associated with health policy challenges, including rapid growth in health care spending. Public hospitals received only minimal public investment and were required to raise revenue by charging patients for services. Patients in turn lacked risk pooling arrangements, so the burden of out-of-pocket expenditure increased [11,12].

Since 1980, China’s regulatory framework has allowed private medical practice, to help alleviate the shortage of public sector medical services. Private sector delivery grew but remained limited; by 1990, only 3.3 percent of health workers at the township or higher levels were in private practice [13]. Although a shortage of data precludes a comprehensive picture of private delivery nationally, it appears concentrated at two polar extremes: on the one hand, “top-end” private providers in China’s largest cities offer advanced medical care with high-tech equipment at very high prices compared to national per capita income; on the other hand, small private clinics and village doctors in towns, villages, and newly urbanized areas offer a mixture of traditional herbal and biomedicine services at low prices to traditionally underserved or vulnerable populations. Some studies suggest that private providers have gradually earned a market share, at least among certain social groups, through lower prices, more flexible hours, better attitudes, and convenience [10]. However, many complaints and lawsuits against private providers allege poor quality, fake drugs, and unqualified staff. Since the early 1990s, laws and rules have set minimum standards of care, but the controversy over the appropriate role of private providers continues [7,10]. Private providers complain that they are unfairly treated compared to their public counterparts across a range of policies, such as purchasing pharmaceuticals, hiring and training personnel, or obtaining contracts for clinical services provided to beneficiaries of social health insurance plans.

These perceptions contributed to the reform debate that culminated in China’s national health reforms, announced in April 2009. These reforms open up policy space for incorporating private providers into the provider network for social insurance and population health services.

#### Community health services in urban china

Traditionally, health care in urban areas has primarily consisted of hospitals, which run large outpatient departments. Beginning in the 1990s, policymakers began to establish a system of community-based health service centers in urban areas to meet the needs associated with China’s demographic and epidemiological transitions and rapid urbanization. Unclear policy priorities and lack of fiscal support led to slow development: by 2006, community health service organizations – centers and stations^a^[2] – only accounted for 8.9 percent of total health care organizations and 2.7 percent of health workers in urban areas [14]. Since 2006, China has encouraged the development of community-based health services by providing financial subsidies for some services, extending health insurance coverage to qualified community providers, and encouraging private providers to join the community health service network, while nevertheless maintaining government-owned providers as the dominant ownership form [15]. According to a 2007 survey of 28 large cities, about one in five urban community health service organizations is privately owned [14].

One goal of China’s current health reforms is to guarantee all Chinese access to basic health care, including having a community health provider within walking distance. The 2009 national health reforms encourage private sector participation, while leaving the detailed implementation up to local authorities. Cities such as Shanghai, Beijing, Wuxi, Foshan, Tieling, and Jixi have started to incorporate private providers into contracting arrangements for primary and preventive health services, but little information is available about the process and perceived success of these initiatives [16].

In June 2008, authorities in Weifang, Shandong, used a public bidding process to select and contract with both government-owned and private facilities for preventive health services and selected clinical services. Our case study examines the implementation of this reform.

### Case study setting and data

#### Case study setting

Weifang is in coastal Shandong province. GDP per capita in 2008 was $4112, and urban residents’ income per capita was $2307.50, close to the national average. In 2008, Weifang urban residents’ medical expenditure per capita was $171.38 [17]. To provide some comparative context for understanding Weifang’s reforms, we also discuss the process of contracting for primary care in City Y, which is near Weifang. In 2008, City Y’s GDP per capita was $7171, urban resident income per capita was $2845, and urban resident medical expenditure per capita was $148.38 [18].

From the mid-1990s to 2004, Weifang was one of the few cities in China that attempted to establish a network of primary care providers and reduce reliance on hospital-based services. All included primary care organizations were government-owned and managed [19]. Local authorities felt that this initiative had achieved only limited success, and that attempts to further strengthen the primary care delivery network would benefit from fresh approaches. Therefore in 2008, the city government decided to promote community health services by contracting with public and private providers on equal terms.

#### Weifang’s contracting policies

An important objective of the 2008 policy change was to influence private providers’ revenue structure, giving incentives to provide the newly contracted preventive health services (Table 1) as well as enabling them to compete on a more equal footing for patients seeking curative primary care services.

**Table 1:** Pay-for-performance in Chinese urban primary care: Criteria for community health station performance scores in Weifang, Shandong

**Items**	**Weights (W)**	**Assessment indicators (A)**	**Scoring (S)**
1. Understanding of the community served	3	Evaluation	grade 0 to 3
2. Community members’ health records	10	X_2_/R	A_2_*W_2_
3. Health education	10	X_3_/R	A_3_*W_3_
4. Infectious disease reporting	3	Evaluation	grade 0 to 3
5. Chronic disease management *	20	X_5_/R_5_	A_5_*W_5_
6. Maternal health care	5	X_6_/R_6_	A_6_*W_6_
7. Child health care	5	X_7_/R_7_	A_7_*W_7_
8. Elderly health care	5	X_8_/R_8_	A_8_*W_8_
9. Health care for the disabled	5	X_9_/R_9_	A_9_*W_9_
10. Public emergency response	4	Evaluation	grade 0 to 4
11. Residents’ satisfaction	30	X_11_/R_11_	A_11_*W_11_
Total	100	----	∑ *S*

*The patient cases which are supposed to be managed include patients with diagnosis of hypertension, diabetes, coronary heart disease, and stroke.

Note: Subscripts refer to the item number. For example, A_2_ refers to the assessment indicator for item 2, and W_2_ denotes weight given to item 2 in the overall assessment score. The assessment indictor is the percentage of the relevant population in the provider’s catchment area that the provider has served (except for three items that resemble public goods, which are evaluated by a group of supervisors). For example, X_2_ is the number of residents who have health records created and maintained by the Community Health Station (CHS); R is the number of residents who live in the community served by the CHS. Similarly, X_3_ is the number of residents with access to health education materials; X_5_ and R_5_ are the numbers of treated and suspected chronic disease cases in the community; X_6_ and R_6_ are the number of women the CHS provides with systematic health care and the number of women living in the community, respectively; X_7_ and R_7_ are the number of children covered by physical monitoring and guideline treatment and the number of children living in the community; X_8_ and R_8_ respectively represent the number of elderly provided with home visits and physical monitoring and the number of elderly people living in the community; X_9_ and R_9_ are the number of the disabled residents with access to rehabilitation and home visits and the number of disabled people living in the community; X_11_ and R_11_ are the numbers of interviewees satisfied with the CHS and total number of interviewed residents in the community, respectively.

Specific features of Weifang’s contracting reform included the following:

•Private providers could become part of the community health system through bidding and contracting with the local government.

•Private providers that successfully competed for a contract could apply for financial subsidies for medical equipment, with the stipulation that the equipment purchased with government funds must be returned if the private provider were to withdraw from the community health system.^b^

•Private providers were required to provide preventive health services which would be reimbursed from the government’s budget after an annual evaluation; see Table 1.^c^ In 2008–2009, the maximum payment for preventive and management services was 10 RMB per resident, payable in whole only if the CHS earned a performance score of 90 or higher. The CHS would receive 70 percent (i.e., 7 RMB per resident) if its score was between 80 and 90; 50 percent (i.e., 5 RMB per resident) if the performance score was between 60 and 80; and no subsidies if its score was less than 60. The CHS administrators were aware of this formula before the contracting period started.

•Clinical care provided by contracted private providers would be covered by social health insurance plans, enabling them to compete for insured patients and get equal access to the drugs listed on the new Essential Medication List (EML). The EML medications are jointly procured by the Weifang bureaus of finance and health and distributed directly to CHS throughout the municipality. Each CHS is reimbursed directly for prescribing medications on the list.

•Personnel working at private CHS were included in capacity-building programs of the local health administration that had previously been limited to the employees of government-owned CHS. These programs included (1) training courses and guidelines about qualified health services; (2) feedback and monitoring during routine inspections and evaluations; and (3) technical support from local general hospitals [20-27].

In June 2008, Weifang authorities chose 63 bidders as official community health stations (CHS) to provide the population health and primary care needs of the resident population. Each CHS served between 5,000 and 10,000 residents. 30 were government-owned and 33 were private facilities.^d^ All met standards regarding staffing, space, and equipment.^e^

City Y also contracted with both public (22) and private (44) CHS for preventive services, using the same evaluation criteria (Table 1 and first row of Table 2). However, this was the only revenue source that was available to private providers on the same terms as public providers: private CHS in City Y were not given access to government subsidies for equipment or technical guidance, and were not included in the provider network for social insurance. Thus City Y is similar to many parts of China (including Weifang before 2008) regarding the role of private providers.

**Table 2:** Revenue sources for community health stations before and after the 2008 reform in Weifang, compared to City Y

**Revenue sources**	**Weifang, 2007 (pre-reform)**		**Weifang, 2009 (post-reform)**		**City Y, 2009 (comparison city)**	
	**Public**	**Private**	**Public**	**Private**	**Public**	**Private**
Subsidies for preventive services*^1^	Yes, according to the preventive services provided.	No.	Yes, decided by number of served residents and evaluation based on the contract.	Yes, same as for public CHS.	Yes, decided by number of served residents and evaluation based on the contract.	Yes, same as for public CHS.
**Subsidies for staff training**	No.	No.	Yes, government provides some free training programs.	Yes, same as for public CHS.	Yes.	No.
**Subsidies for personnel**	Yes, per capita budget and payment for retirees’ social insurance.	No.	Yes, same as before.	No.	Yes.	No.
**Subsidies for rental or purchase of land and clinic space**	Yes, but amount differs according to CHS scale and scope.	No.	Yes, same as before.	No.	Yes.	No.
**Subsidies for equipment**	Yes.	No.	Yes, one time 60,000RMB investment.	Yes, one time 60,000RMB investment. Refund to government if CHS withdraws from the CHS network.	Yes.	No.
Subsidies for EML drugs*^2^	N/A (EML drug policy not yet launched.)	N/A (EML drug policy not yet launched.)	Yes, sell at acquisition price to patient; government pays the CHS the original 15% mark up for dispensing EML drugs.	Yes, same as for public CHS	N/A (EML drug policy not yet launched.)	N/A (EML drug policy not yet launched.)
**Fee for service from out-of-pocket payments**	Patients charged according to government-set fixed or “guide” prices.	CHS has autonomy in setting prices.	Same as before.	CHS retains price-setting autonomy, but cannot exceed government “guide” prices.	Patients charged according to government-set fixed or “guide” prices.	CHS has autonomy in setting prices.
Fee for service paid by urban employee insurance *^4^	Covered, but no difference from hospital outpatient care in terms of patient co-payment.	Not covered by the social insurance network.	Covered, and at a more generous rate than hospitals. Patient co-payments are lower than for hospital outpatient visits.	Yes, same as for public CHS.	Covered, but no difference from hospital outpatient care in terms of patient co-payment.	Not covered by the social insurance network.
Fee for service paid by urban residents insurance *^4^	N/A (Urban residents insurance not yet launched.)	N/A (Urban residents insurance not yet launched.)	Covered, and at a more generous rate than hospitals. Patient co-payments are lower than for hospital outpatient visits.	Yes, same as for public CHS	Covered, but no difference from hospital outpatient care in terms of patient co-payment.	Not covered by the social insurance network.

*1. The subsidies are decided by (a) the evaluation score as described in Table 1; and (b) the number of served residents. In 2008 and 2009, the per capita budget for public health services was 10RMB. CHS with a score above 80 got 100% of the budget; CHS scoring between 70 and 80 got 90% of the budget; CHS scoring between 60 and 70 got 80% of the budget; CHS scoring between 50 and 60 got 60% of the budget; and CHS scoring under 50 got no subsidies.

*2. The 70 drugs listed on the Essential Medicine List must be sold to patients at the acquisition price; if prescriptions from the EML represent more than 30% of all prescriptions, the CHS receives a subsidy from the government equivalent to 15% of the drug price.

*3. The chronic diseases for which the CHS can be reimbursed by health insurance for associated outpatient expenses include stroke, diabetes, chronic viral hepatitis, and autoimmune hepatitis.

*4. For the service items covered by insurance, CHS are reimbursed by insurers and patient copayments.

All CHS in both cities are non-profit and thus do not pay taxes. Table 2 describes the revenue sources of public and private providers before and after the reform in Weifang, compared to City Y.

### Data collection

We collected data from three primary sources: administrative records at the municipal and provincial levels; a household survey of 1013 community residents in Weifang and 681 residents in City Y; and a provider survey of 1298 staff at CHS in both Weifang and City Y.

For the household survey, a locally contextualized questionnaire was developed in Chinese, based on the Primary Care Assessment Tool (PCAT) of Johns Hopkins University [28]. All communities were divided into two groups according to ownership of the CHS designated to serve patients in that area: residential communities served by a government-owned CHS (Pub-C hereafter) and communities served by a privately-owned CHS (Pri-C hereafter). A total sample of 1013 households was selected from the total number of households served by the CHS, by first randomly selecting CHS in each ownership group and then interviewing a quasi-random sample of residents in those neighborhoods. In November 2009, trained interviewers carried out the field work with supervision. For quality assurance, five percent of households were re-investigated by academic supervisors. Interviewees voluntarily participated in the survey with the assurance of confidentiality. The study was conducted according to policies of the Ministry of Health of China and was approved by the Institutional Review Board at Stanford University.

We supplement the household survey data with data from anonymous questionnaires that were distributed to all staff members in both public and private CHS in both cities and collected in a sealed envelope. We supplemented the quantitative data with one-on-one, in-depth interviews with key informants, including local health and finance officials in charge of contracting with and paying CHS institutions.

## Methods

For descriptive and comparative analysis between public and private providers, univariate, bivariate and multivariate analyses were performed using SPSS for Windows, version 11.0 and Stata 9.

Comparing providers of different ownership forms is inherently complicated by the differences in their characteristics. For example, smaller providers may have systematically lower performance if there are economies of scale and scope in performance, and private providers tend to be systematically smaller than government-owned providers. If mean performance scores are lower for private CHS than public CHS, to what extent is this difference because private CHS are smaller, rather than because they are private? To answer such questions, multivariate regressions that control for potentially confounding factors are useful.

We run descriptive regressions of the following form:

$$\begin{array}{l} y_{\text{it}}=\lambda +\beta_{i}X_{\text{it}}+\beta_{\text{Own}}\text{Privat}e_{i}+\beta_{\text{City}}\text{Weifan}g_{i}\\ \;+\beta_{\text{Own}\_W}\left(\text{Private}*\text{Weifang}\right)+u_{\text{it}}\text{,} \end{array}$$

Where *y*_*it*_ is the dependent variable of interest, such as the performance score for provider *i* in year *t*; *λ* is a constant; ***X*** represents a vector of observed characteristics of the provider; *β*_*i*_ is the set of estimated coefficients for the characteristics in vector ***X***; *Private*_*i*_ denotes private ownership; *Weifang*_*i*_ is a dummy variable equal to 1 if provider *i* is located in Weifang, rather than City Y; and *u*_*it*_ denotes idiosyncratic errors. The primary estimated coefficient of interest is *β*_*own_W*_, representing the difference in the dependent variable between private and public providers in Weifang, compared to *β*_*own*_ for City Y, controlling for other observable characteristics. If the sum of the estimated coefficients for *Private*_*i*_ and Private * Weifang is statistically indistinguishable from zero, then there is no statistically significant difference in performance between public and private CHS in Weifang, after controlling for the fact that smaller CHS tend to have lower performance scores in both Weifang and City Y. Data limitations preclude any multi-year analyses; all our regressions are cross-sectional.

For example, consider our analysis of factors associated with CHS performance scores in 2009. In this analysis, t = 2009 and *y*_*it*_ is the 2009 performance score for CHS *i*; the ***X*** vector includes such characteristics of the CHS as number of beds, number of staff, fixed assets, a dummy variable for being in the social health insurance network as an appointed provider, and a dummy variable for implementing the policy of separating prescribing and dispensing.

One empirical challenge is that several of the CHS characteristics are correlated (e.g., providers with more beds also have more staff and are more likely to be included in the insurance network as appointed providers). To enable qualitative statements about CHS of different ownership form *with otherwise similar characteristics*, we present results for multiple specifications of each regression, alternatively including different sets of explanatory variables. We confine our discussion of the primary estimate of interest—*β*_*own_W*_ —to the case in which the estimate is relatively unchanged across these difference specifications. Stability of the estimate across different empirical specifications reflects robustness to different ways of controlling for the observable differences among providers.

These multivariate regressions are not intended to constitute an impact evaluation of the contracting reforms in Weifang. Several limitations of the available data preclude a research design that could evaluate impact or disentangle causality. For example, we lack baseline data for pre-reform trends in Weifang and the comparison group; and two years is probably too short a time frame for an evaluation of most dimensions of provider performance. Rather than an impact evaluation, the analyses document differences between ownership forms, controlling for observable factors (such as size). Such analyses enable statements of the form “public and private CHS differ in size and staffing, but once we control for these differences, public and private CHS no longer statistically differ in their performance scores in Weifang in 2009.”

We also use the survey data of CHS personnel to explore the factors associated with staff satisfaction and performance scores of community health stations in Weifang and City Y. In those regressions, in addition to the performance score *y*_*it*_ for t=2009, we define a second dependent variable to be equal to 1 if the staff member reports being satisfied with his or her job. For the latter limited dependent variable, we use a logit analysis. The ***X*** vector of observed characteristics of staff member *i* includes a dummy variable if male; age and aged squared; years of work experience at the CHS; and dummy variables for educational attainment and job position (i.e., general practitioner, nurse, manager, pharmacist, technician).

A third and final set of multivariate analyses describes the association between resident characteristics and their utilization of CHS services. Suppose that we find, as we do, that on average more residents served by private CHS than public CHS say they are willing to do routine examinations at the CHS. Is this because private CHS provide better quality services than their public counterparts do, or because the residents in private-CHS communities tend to use community services more often than hospital outpatient departments? To address this question, we run logistic regressions of the following form:

$$Y_{i}=\lambda +\beta_{i}X_{i}+u_{i}$$

The dependent variable *Y*_*i*_ is a dichotomous variable about the health service utilization of individual *i*. For example, to estimate the probability of being served by a privately owned provider, *Y*_*i*_ is equal to 1 if the resident lives in a community served by a private CHS; to estimate the probability that the resident is willing to use the community provider (rather than go to a hospital outpatient department), *Y*_*i*_ is equal to 1 if the resident says he or she is willing to do a routine health exam at the neighborhood CHS, or is willing to visit the CHS for first-contact care when feeling ill. The dichotomous explanatory variables *X*_*i*_ include male; uninsured; self-reported poor health status; low education; and low income (i.e., monthly income below 800 RMB). These regressions allow us to see whether utilization preferences are systematically associated with the individual socioeconomic characteristics of community residents. Standard errors are clustered at the CHS level.

## Results

### Performance scores for contracted services

The average 2009 performance scores for the contracted population health services in Weifang were higher than in City Y, with no statistically significant difference by ownership (Table 3). In Weifang, the average performance scores for public and private CHS were 83.75 and 82.76 respectively. In contrast, City Y’s overall performance was lower and there was a significant gap between the scores of public and private CHS (78.22 for public and 70.35 for private CHS). It is unclear from these scores whether the contracting initiative in Weifang was cause or effect; Weifang’s authorities turned out to have been justified in their assumption that private CHS could provide community services at a comparable level as their government-owned counterparts. The gap in performance between public and private CHS in City Y, in turn, suggests that either the lack of supportive policies contributed to a gap in performance by ownership; or City Y authorities were justified to think that private CHS in City Y were not prepared and willing to meet the same targets for preventive and primary care services as the public CHS were; or both.

**Table 3:** Comparing public and private community health stations before and after Weifang’s reform, compared to City Y

**Indicators (Mean)**	**Weifang**		**Y**	
	**Public**	**Private**	**Public**	**Private**
	(30)	(26)	(23)	
Performance score (evaluation by supervisors regarding contracted preventive services)				
2007	----	----	----	----
2009	83.75	82.76	78.22	70.35
Value of fixed assets (RMB)				
2007	----	----	----	----
2009	168.50	42.59	29.83	22.52
Average number of staff members				
2007	10.50	7.88	----	----
2009	9.36	8.65	11.13	8.11
Government subsidies as a percentage of total revenue				
2007	15.57	----	----	----
2009	41.72	50.22	36.42	4.81
Expenditure per visit (RMB)				
2007	35.79	12.26	----	----
2009	29.27	14.42	59.54	39.91
Patient visits per staff member				
2007	465.21	1245.23	----	----
2009	483	1149	686.67	941.68
Home visits by per staff member				
2007	24.20	29.47	----	----
2009	19.49	39.47	10.62	29.17
Number of home beds per staff member				
2007	1.40	12.43	----	----
2009	0.64	17.72	3.98	8.50
Referrals to inpatient treatment (%)				
2007	0.57	0.27	----	----
2009	0.49	0.39	1.49	0.06

Differences in performance scores between CHS of different ownership form, and among CHS of the same ownership form, could be partly explained by differences in the underlying capacity of the provider organizations. Particularly if there are fixed costs in establishing administrative systems for monitoring community services and economies of scale and scope in outreach, then larger CHS will have an advantage in meeting the thresholds for acceptable (reimbursable) performance for the various population health services.

We find that in both Weifang and City Y, the average value of fixed assets of public CHS is larger than that of private CHS. Interviews confirm that this discrepancy arises primarily because government providers own their facilities, whereas most private providers lease their facilities. Private CHS in Weifang increased the average number of staff to meet the staffing criteria for community service network providers. Most of the public CHS already had enough staff to meet the criteria.

During the implementation of the contracting reform, providers experienced a significant change in their revenue structure. Information from interviews confirmed that the revenue change was especially salient for private providers, because they had never before received public financing. For public CHS in Weifang, the percentage of subsidies out of total revenue increased from 13 percent in 2007 to 42 percent in 2009. Since many private CHS did not exist or were not part of any standardized administrative record system in 2007, we do not have comparable pre-reform data for private CHS. We do know that by 2009, fully half of reported revenue for private CHS in Weifang came from government subsidies. This large proportion of income reflects both the new subsidies for preventive services and equipment, and the lower market share of private CHS in curative services.

The significance of the contracting policies in shaping this change in revenue structure for primary care providers is evident when comparing Weifang to City Y. In 2009, private CHS in City Y earned less than 5 percent of their revenue from government subsidies. City Y paid subsidies only for the contracted preventive services, and private providers in Y were less likely to receive those subsidies in full because of lower performance scores compared to government-owned CHS in City Y; see Table 4.

**Table 4:** Distribution of performance scores for community health stations in Weifang and City Y in 2009

		**Score **_**≥**_**90 (got total budget)**	**Score between 80 and 90 (got 70% of budget)**	**Score between 60 and 80 (got 50% of budget)**	**Score below 60 (no subsidies)**
Weifang	Government	6 (91.00)	18 (83.12)	9 (77.12)	0
	Private	3 (92.80)	21 (82.96)	7 (76.79)	0
City Y	Government	2 (93.30)	6 (84.53)	16 (73.97)	0
	Private	0	7 (85.49)	25 (72.36)	8 (50.81)
TOTAL	Government	8 (91.58)	24 (83.47)	25 (75.11)	0
	Private	3 (92.80)	28 (83.59)	32 (73.33)	8 (50.81)
	Total	11 (91.91)	52 (84.54)	57 (74.11)	8 (50.81)

Notes: Each cell represents the number of community health stations in that category, with the associated average score, conditional on being in that category, reported in parentheses.

In discussing the performance of primary care providers, it is important not only to consider the average performance score, but also the distribution of the performance scores [1]. Table 4 summarizes this distribution for both cities in 2009. Compared to Weifang, the dispersion of performance scores is greater in City Y, where the scores spanned the entire gamut from highest (fully reimbursed) to worst (unreimbursed) performance. The distribution of City Y private providers’ scores is of similar dispersion but shifted downward in mean value, compared to the distribution of scores for City Y government providers (or compared to Weifang providers of either ownership form). Only 8 of 120 providers scored below 60 and were thus denied any subsidies; all 8 of those CHS were private providers in City Y.

The expenditure per visit in public CHS was higher than in private CHS in both Weifang and City Y. This crude metric of spending (obtained by dividing total reported revenue by number of visits) reflects both the mix of services provided as well as the use of resources per visit. With government-owned CHS providing more clinical services and referrals to hospitals, it is not surprising that resource use per visit would be higher.^f^

Private CHS provided more visits per staff member than public CHS did, with a larger ownership difference in Weifang than City Y. This ownership difference in visits per staff member in Weifang was narrower in 2009 than before reform.

That private providers actively sought out niches in the market is evident from ownership differences in community-oriented clinical services: private CHS were more active in providing home visits to temporarily immobile patients, or regular home visits for the elderly and other patients with special needs. Public providers referred more often to specialty care than private providers did, but the ownership-associated gap in referrals was smaller in Weifang in 2009 than before reform.

Table 5 presents the factors associated with CHS performance scores in a multivariate context to control for potentially confounding observable factors. The results confirm that while private CHS scored lower than public CHS, the difference disappeared for Weifang, where there is no statistically significant difference in performance between public and private CHS (see Additional file 1: Appendix Table S2 for the corresponding F tests).

**Table 5:** Factors associated with CHS performance scores, 2009

**Dependent variable: 2009 performance score**					
	**(1)**	**(2)**	**(3)**	**(4)**	**(5)**
Private	−6.212	−7.030	−7.026	−5.548	−5.311
	(3.651)*	(3.615)*	(3.506)**	(3.556)	(3.505)
Weifang	5.578	6.234	6.313	6.368	6.994
	(2.273)**	(2.137)***	(1.999)***	(2.351)***	(2.223)***
Private*Weifang	7.480	7.585	7.273	6.789	6.035
	(3.940)*	(3.885)*	(3.709)*	(3.874)*	(3.809)
Clinic space (m^2^)	0.007	−0.000	0.000		
	(0.004)*	(0.001)	(0.001)		
Value of fixed assets	−0.023			−0.007	−0.007
	(0.014)			(0.008)	(0.007)
Number of beds	0.296	0.330	0.352	0.248	0.277
	(0.280)	(0.267)	(0.249)	(0.268)	(0.264)
Number of staff members	0.206	0.147	0.120	0.362	0.323
	(0.194)	(0.168)	(0.158)	(0.205)*	(0.179)*
Dummy for medical insurance appointed health stations	−0.407	−0.852		−1.135	
	(3.047)	(2.932)		(3.059)	
Dummy for implementing the policy of separating prescribing from dispensing	1.939	−0.754		0.604	
	(2.608)	(2.229)		(2.226)	
Constant	70.534	72.637	72.083	70.907	70.283
	(3.793)***	(3.755)***	(2.693)***	(3.770)***	(2.910)***
Observations	90	100	106	92	94
Adjusted R-squared	0.16	0.18	0.21	0.15	0.18

Notes: 1) Robust standard errors in parentheses; 2) ***, **, * refer to 1%, 5% and 10% statistical significance level, respectively.

### Data from household surveys

Table 6 shows the socioeconomic status of interviewed households. Residents in districts served by public CHS (the Pub-C group) are generally of higher socioeconomic status than the residents served by private CHS (Pri-C group). Compared to Pub-C, private CHS serve catchment areas with fewer formal employees and more unemployed residents, retirees, and farmers. Private CHS catchment areas also have a significant minority of residents in the lowest income group, and residents with no more than an elementary school education. These differences are not surprising, since public CHS developed first in the central urban neighborhoods, whereas private CHS tend to be located in suburbs or newly urban areas where the majority of residents are domestic immigrants and former farmers.

**Table 6:** Profile of household respondents

**Socio-economic variables (%)**	**Respondents**				**Respondents who visited the facility**			
	**All (n=1013)**	**Pub-C (n=445)**	**Pri-C (n=568)**	**Pri-Pub Difference**	**All (n=681)**	**Pub-C (n=299)**	**Pri-C (n=382)**	**Pri-Pub Difference**
**Gender**								
Female	60.1	59.6	60.6	1.01	59.5	59.2	59.7	0.49
Male	39.3	40.0	38.7	−1.27	40.2	40.5	40.1	−0.42
**Occupation**								
Civil Servant	2.9	2.9	2.8	−0.10	2.6	3.0	2.4	−0.65
Formal employee	35.4	41.8	30.5	−11.34***	36.6	44.1	30.6	−13.52***
Informal employee	10.6	10.8	10.4	−0.40	9.3	10.0	8.6	−1.39
Unemployed	7.3	5.2	9.0	3.81**	7.6	4.0	10.5	6.46***
Farmer	2.9	1.8	3.7	1.90*	2.3	2.3	2.4	0.01
Retired	27.1	24.3	29.4	5.13*	29.4	24.1	33.5	9.43***
Student and preschool children	1.8	1.8	1.8	−0.04	1.0	1.0	1.0	0.04
Others	11.5	11.2	11.6	0.38	10.6	11.0	10.2	−0.83
**Education**								
Elementary school or lower	13.7	11.2	15.7	4.43**	13.2	11.0	14.9	3.88
Middle school	29.5	27.9	30.8	2.94	28.6	26.4	30.4	3.95
High school	29.0	30.8	27.6	−3.15	29.2	31.1	27.7	−3.35
Junior college	16.6	16.9	16.4	−0.48	17.3	17.7	17.0	−0.71
College	9.8	11.0	8.8	−2.21	10.4	11.7	9.4	−2.28
Master degree	0.8	1.6	0.2	−1.40**	0.6	1.3	0.0	−1.34**
Doctorate	0.0	0.0	0.0	0.00	0.0	0.0	0.0	0.00
**Monthly income**								
<400	0.0	0.0	0.0	0.00	0.0	0.0	0.0	0.00
400-800	12.5	8.8	15.5	6.73***	11.0	7.7	13.6	5.92**
800-1200	24.6	25.2	24.1	−1.05	26.6	27.1	26.2	−0.91
1200-1600	20.1	19.3	20.8	1.45	19.8	18.4	20.9	2.55
1600-2400	15.6	17.5	14.1	−3.44	16.6	19.7	14.1	−5.60*
2400-3000	6.5	9.0	4.6	−4.41***	5.9	9.4	3.1	−6.22
3000-4000	5.4	7.0	4.2	−2.74*	5.0	6.4	3.9	−2.43
4000-6000	2.8	2.7	2.8	0.12	3.7	3.7	4.7	1.00
6000+	1.4	0.4	2.1	1.66**	1.9	0.3	3.1	2.81***
Did not report	10.9	9.9	11.6	1.73	9.3	7.0	11.0	3.97*
**Health insurance coverage**								
Urban employee	16.4	11.5	20.2	8.79***	16.9	11.0	21.5	10.43***
Urban residents	24.6	29.4	20.8	−8.66***	26.6	33.4	21.2	−12.24***
NCMS	1.9	2.0	1.8	−0.26	1.6	2.3	1.0	−1.29
Commercial insurance	3.7	4.9	2.6	−2.30*	2.8	3.3	2.4	−0.99
OOP	52.6	51.2	53.7	2.46	51.5	49.2	53.4	4.24
**Self reported health status**								
Excellent	9.4	9.4	9.3	−0.11	9.1	8.7	9.4	0.73
Very good	34.0	39.8	29.4	−10.37***	32.2	36.8	28.5	−8.26**
Good	34.3	31.9	36.1	4.18	35.7	35.1	36.1	1.01
Not bad	17.0	13.9	19.4	5.43**	17.2	14.4	19.4	4.99*
Bad	5.1	4.5	5.6	1.14	5.6	4.7	6.3	1.60

Notes: 1) Pub-C refers to a catchment area served by a public (government-owned) CHS; Pri-C refers to a catchment area served by a private CHS; 2) * New Cooperative Medical System; 3) ***, **, * refer to 1%, 5% and 10% statistical significance level, respectively; here we report proportion test (‘prtest’ in Stata) results (P-values) for public-private differences, rather than mean (‘ttest’ in Stata) because the ttest command relies on some distributional assumptions that may not be true for each component; 4) the summation for each category may not equal 100% because of missing values.

### Residents’ attitudes towards CHS and utilization patterns

As shown in Table 7, about two-thirds of residents knew the names or addresses of their neighborhood CHS. 67.2 percent of respondents reported that at least one family member had ever visited the neighborhood CHS. With an average household size of three, this means that at least one in five residents had visited their neighborhood CHS.^g^

**Table 7:** Comparison of residents’ knowledge, attitudes, and preferences regarding their neighborhood community health station

**Socioeconomic variables (%)**	**Respondents**				**Respondents who visited the facility**			
	**All (n=1013)**	**Pub-C (n=445)**	**Pri-C (n=568)**	**Pri-Pub Difference**	**All (n=681)**	**Pub-C (n=299)**	**Pri-C (n=382)**	**Pri-Pub Difference**
**Know the name or address of the neighborhood CHS**								
No	35.3	37.8	33.5	−4.30	21.1	23.4	19.4	−4.04
Yes	64.7	62.2	66.5	4.30	78.9	76.6	80.6	4.04
**At least one family member visited this CHS before**								
No	26.6	27.9	25.5	−2.34	0.0	0.0	0.0	0.00
Yes	67.2	67.2	67.3	0.06	100.0	100.0	100.0	0.00
Not sure/Forgot	5.7	4.9	6.3	1.39	0.0	0.0	0.0	0.00
**Willing to do routine examination at this CHS**								
No	26.7	31.9	22.5	−9.37***	16.6	21.1	13.1	−7.98***
Yes	49.0	42.2	54.2	11.98***	65.5	58.5	70.9	12.41***
Not sure	22.6	25.6	20.2	−5.37*	17.5	20.4	15.2	−5.22*
**First visit this CHS when sick**								
No	24.9	30.8	20.2	−10.54***	12.2	17.1	8.4	−8.68***
Yes	52.0	47.2	55.8	8.62***	69.8	64.5	73.8	9.27***
Not sure	21.6	21.8	21.5	−0.32	18.1	18.4	17.8	−0.59
**Received advertising materials from this CHS**								
Yes	46.1	41.3	49.8	8.48***	56.5	53.2	59.2	5.99
No	34.6	40.0	30.3	−9.72***	29.8	35.1	25.7	−9.46***
Not sure	17.9	18.4	17.4	−1.00	13.7	11.7	15.2	3.48
**Can call responsible doctors at any time when needed**								
Yes	42.8	40.7	44.5	3.87	54.5	52.8	55.8	2.92
No	10.1	9.2	10.7	1.53	9.5	8.4	10.5	2.11
Not sure	45.3	49.7	41.9	−7.76**	35.8	38.5	33.8	−4.69
**Do providers at this CHS know the health status of your family members?**								
Very well	5.6	5.6	5.6	0.02	7.3	7.7	7.1	−0.62
Yes	32.1	31.9	32.2	0.31	42.7	41.8	43.5	1.65
No	38.4	36.9	39.6	2.76	28.8	26.1	30.9	4.80
Not sure	22.2	25.4	19.7	−5.67*	21.1	24.4	18.6	−5.83*

Notes: 1) Pub-C refers to a catchment area served by a public (government-owned) CHS; Pri-C refers to a catchment area served by a private CHS; 2) * New Cooperative Medical System; 3) ***, **, * refer to 1%, 5% and 10% statistical significance level, respectively; here we report proportion test (‘prtest’ in Stata) results (P-values) for public-private differences, rather than mean (‘ttest’ in Stata) because the ttest command relies on some distributional assumptions that may not be true for each component; 4) the summation for each category may not equal 100% because of missing values.

Comparing public and private CHS, we find that more residents in Pri-C (51.1 percent) had received information from their neighborhood CHS than those in Pub-C (41.4 percent). Residents served by a private CHS were also more likely to report willingness to visit the CHS for a routine examination or for first-contact care when ill. Respondents who had previously visited the CHS were more willing to do so again than residents who had never visited the CHS, and the gap favoring private facilities remains. These results may indicate that private providers serve communities more likely to visit a CHS than a hospital for first-contact care, and that private CHS probably offered relatively stronger incentives to staff members for marketing their services than their public counterparts. There were no significant differences between public and private CHS regarding residents’ perceived ability to reach doctors by phone and providers’ knowledge about their families.

A simple descriptive regression (Table 8) confirms differences in the communities served by public and private CHS, and in residents’ patterns of utilization. The first column shows that residents in the communities served by private CHS were more likely to be uninsured and to report poor health, compared to residents in public CHS communities (Pub-C). Controlling for other factors, uninsured patients were less likely to have visited a CHS, presumably because they were less likely than insured residents to seek formal treatment for illness or preventive services. When controlling for individual characteristics, we find that residents were more willing to do a routine health exam at their neighborhood CHS if they were of low socioeconomic status (as measured either by education or income) and if the CHS was private.^h^ Results regarding resident willingness to visit the CHS for first-contact care are similar to those for routine exams: insured patients, those of low socioeconomic status, and those served by private CHS were more likely to visit the CHS when ill. These patterns are consistent with the long-standing perception of CHS in China as providing convenient but lower quality care than hospital outpatient departments.

**Table 8:** Multivariate analysis of residents’ usage of community health station services (logistic regressions)

**Dependent variables:**	**Prob(Served by Pri_C)**	**Prob(visited CHS in any district)**	**Probability that resident is willing to do routine health exam at neighborhood CHS**				**Probability that resident is willing to visit the neighborhood CHS as first contact if ill**	
	**All CHS (all districts)**	**All CHS (all districts)**	**All CHS (all districts)**	**Public CHS**	**Private CHS**	**All CHS**	**All CHS**	**All CHS**
	**All respondents**	**All respondents**	**All respondents**	**Respondents who previously visited the CHS**	**Respondents who previously visited the CHS**	**Respondents who previously visited the CHS**		
	(1)	(2)	(3)	(4)	(5)	(6)	(7)	(8)
Male	-0.020	0.192	0.121	0.276	-0.098	0.083	-0.093	-0.190
	(0.168)	(0.135)	(0.140)	(0.250)	(0.245)	(0.173)	(0.116)	(0.175)
Uninsured	0.783	-0.349	-0.258	-0.788	0.743	0.114	-0.320	-0.113
	(0.300)***	(0.133)***	(0.130)**	(0.374)**	(0.185)***	(0.253)	(0.150)**	(0.232)
Self reported poor health	0.344	0.139	-0.007	-0.038	-0.153	-0.060	-0.004	-0.131
	(0.222)	(0.180)	(0.177)	(0.272)	(0.319)	(0.192)	(0.120)	(0.163)
With no higher than compulsory education	0.015	-0.124	0.139	0.861	0.151	0.419	0.016	0.113
	(0.326)	(0.213)	(0.192)	(0.400)**	(0.394)	(0.274)	(0.205)	(0.310)
Monthly income lower than 800RMB	0.346	0.050	0.418	-0.272	1.231	0.497	0.366	0.525
	(0.437)	(0.316)	(0.230)*	(0.597)	(0.569)**	(0.425)	(0.198)*	(0.386)
Neighborhood served by private CHC		0.180	0.499			0.434	0.405	0.412
		(0.302)	(0.412)			(0.489)	(0.378)	(0.491)
Constant	-0.109	0.909	-0.417	0.108	0.517	0.115	-0.070	0.627
	(0.546)	(0.245)***	(0.309)	(0.394)	(0.398)	(0.379)	(0.282)	(0.403)
Observations	918	866	918	282	347	629	918	629

Notes: 1) Standard errors in parentheses are clustered at the CHS level; 2) ***, **, * refer to 1%, 5% and 10% statistical significance level, respectively.

### Residents’ evaluations

Responses to survey questions about primary health care are shown in Table 9, conditional on the respondent having visited a CHS. In contrast with general residents (who may not have ever visited the CHS before and who generally were not aware of the CHS hours of operation^i^), respondents who had visited their neighborhood CHS were more knowledgeable about and favorably disposed toward CHS services.

**Table 9:** Comparison of residents’ evaluations of their neighborhood community health station, for residents who had visited the station

**Questions and Answers (%)**	**Definitely**	**Probably**	**Probably not**	**Definitely not**	**Not sure/do not remember**
**Know this CHS to be open on weekends**					
all (N=681)	82.1	7.8	1.6	1.3	6.8
pub (N=299)	81.9	8.0	0.7	1.0	8.4
private (N=382)	82.2	7.6	2.4	1.6	5.5
Pri-Pub Difference	0.26	−0.44	1.69*	0.57	−2.86
**Know this CHS to be open until 8:00 PM**					
all (N=681)	67.1	13.7	3.7	2.5	12.6
pub (N=299)	60.5	13.4	5.4	4.0	16.7
private (N=382)	72.3	13.9	2.4	1.3	9.4
Pri-Pub Difference	11.72***	0.50	−3.00**	−2.70**	−7.30***
**Enough time talking about patients’ medical problems and related concerns**					
all (N=681)	51.0	26.3	13.2	5.0	3.8
pub (N=299)	48.2	30.1	15.1	1.7	4.7
private (N=382)	53.1	23.3	11.8	7.6	3.1
Pri-Pub Difference	4.98	−6.80*	−3.27	5.92***	−1.54
**Understand patients’ situation when patients have difficulty in paying for drugs or other health expenditures**					
all (N=681)	18.6	23.5	25.4	14.1	17.5
pub (N=299)	12.7	26.8	25.4	14.7	20.4
private (N=382)	23.3	20.9	25.4	13.6	15.2
Pri-Pub Difference	10.59***	−5.81*	−0.03	−1.10	−5.22*
**Know patients’ current prescriptions or ask when the patient visits**					
all (N=681)	51.4	28.5	9.1	4.6	5.9
pub (N=299)	46.2	34.8	8.0	4.3	6.7
private (N=382)	55.5	23.6	9.9	4.7	5.2
Pri-Pub Difference	9.34**	−11.22***	1.92	0.36	−1.45
**Able to change CHS if patients want**					
all (N=681)	20.9	23.2	23.2	17.0	15.1
pub (N=299)	22.4	25.4	26.8	13.4	12.0
private (N=382)	19.6	21.5	20.4	19.9	17.5
Pri-Pub Difference	−2.77	−3.95	−6.34*	6.52**	5.50**
**Discuss with patients other possible providers for treating the patients’ medical condition**					
all (N=681)	14.0	22.5	10.6	9.4	8.8
pub (N=299)	14.4	22.1	11.4	9.7	11.0
private (N=382)	13.6	22.8	9.9	9.2	7.1
Pri-Pub Difference	−0.77	0.70	−1.42	−0.54	−3.97
**Help patient to make appointment**					
all (N=681)	8.7	12.5	14.0	17.5	12.6
pub (N=299)	4.3	11.4	16.1	21.7	15.1
private (N=382)	12.0	13.4	12.3	14.1	10.7
Pri-Pub Difference	7.69***	1.98	−3.75	−7.60**	−4.32
**Knowing patients’ results after patient visited a hospital**					
all (N=681)	15.9	15.1	15.3	10.3	8.7
pub (N=299)	14.7	15.4	17.7	9.7	11.0
private (N=382)	16.8	14.9	13.4	10.7	6.8
Pri-Pub Difference	2.04	−0.46	−4.37	1.03	−4.23
**Discuss with patients after they come back from other hospitals**					
all (N=681)	17.9	17.2	11.2	11.0	7.9
pub (N=299)	18.7	16.4	14.4	9.7	9.4
private (N=382)	17.3	17.8	8.6	12.0	6.8
Pri-Pub Difference	−1.45	1.41	−5.74**	2.34	−2.56
**Give suggestions on healthy and unhealthy foods and the benefits of enough sleep**					
all (N=681)	53.9	30.5	6.5	4.8	3.7
pub (N=299)	48.5	33.8	8.0	5.4	4.0
private (N=382)	58.1	28.0	5.2	4.5	3.4
Pri-Pub Difference	9.62**	−5.77	−2.79	−0.90	−0.61
**Give suggestions on home safety such as storage of drugs and chemicals**					
all (N=681)	27.0	24.7	19.8	13.7	14.2
pub (N=299)	15.1	29.1	21.4	19.4	14.7
private (N=382)	36.4	21.2	18.6	9.2	13.9
Pri-Pub Difference	21.34***	−7.89**	−2.82	−10.24***	−0.84
**Ask questions on possible diseases or other health concerns of your family**					
all (N=681)	32.9	41.1	11.6	6.2	7.8
pub (N=299)	25.1	47.5	13.7	5.7	8.0
private (N=382)	39.0	36.1	9.9	6.5	7.6
Pri-Pub Difference	13.92 ***	−11.37***	−3.76	0.86	−0.44
**Provide home visits**					
all (N=681)	36.0	15.7	8.1	21.9	17.9
pub (N=299)	37.8	15.1	9.0	19.1	19.1
private (N=382)	34.6	16.2	7.3	24.1	17.0
Pri-Pub Difference	−3.24	1.18	−1.70	5.02	−2.05
**Ask patients to fill out a questionnaire to better understand patient demand for services**					
all (N=681)	31.3	23.5	8.8	17.5	18.5
pub (N=299)	20.1	29.4	11.0	18.7	20.7
private (N=382)	40.1	18.8	7.1	16.5	16.8
Pri-Pub Difference	19.99***	−10.58***	−3.97*	−2.24	−3.98
**Ask residents to fill out a questionnaire to better understand health issues in the served community**					
all (N=681)	28.8	26.9	8.2	16.4	19.2
pub (N=299)	21.1	29.4	10.7	16.7	22.1
private (N=382)	34.8	24.9	6.3	16.2	17.0
Pri-Pub Difference	13.75***	−4.56	−4.42**	−0.49	−5.06

Notes: 1) ***, **, * refer to 1%, 5% and 10% statistical significance level, respectively; here we report proportion test (‘prtest’ in Stata) results (P-values) for public-private differences rather than mean (‘ttest’ in Stata) because the ttest command relies on some distributional assumptions that may not be true for each component; 2) the summations for each category may not equal 100% because of missing values.

There remains significant room for improvement among all CHS. Only about half of patients said that CHS providers spent enough time talking with them about their medical problems, were familiar with their current prescriptions, or asked about their prescriptions during the visit. Fewer than half of patients reported that their doctors definitely or probably would (a) help make an appointment at a referral hospital, or (b) know the results of the hospital treatment during a follow-up visit at the CHS.

Comparing public and private CHS, a small statistically significant difference favoring private CHS is evident when residents were asked whether providers had given suggestions on healthy and unhealthy foods and the benefits of enough sleep; talked with them about household safety measures such as proper storage of chemicals; asked them to fill out a questionnaire to better understand community needs; and understood patients’ situation when patients had difficulty paying their medical bills.

Thus, although household survey respondents generally gave low evaluations of CHS, residents with experience at CHS were more knowledgeable and favorable about CHS, with more favorable responses for private CHS on the few dimensions where there were ownership differences. It would, however, be premature to conclude that private CHS did better than public facilities in terms of family-centered and community-oriented primary health care, because these differences are small and may partly reflect the socioeconomic status of the residents. Furthermore, local health officials gave contradictory reports during our in-depth interviews (i.e., that public health stations seemed more serious and diligent about finishing the required public health tasks, including patient and community questionnaires). Moreover, among general surveyed residents (as opposed to those who had visited the CHS), more respondents in Pub-C than in Pri-C thought their doctors definitely or probably could help them make appointments at referral hospitals. This difference may reflect the fact that most public CHS are run by public hospitals, and therefore can readily refer patients for inpatient services.

### Staff in public and private community health stations

Table 10 reports the socioeconomic characteristics and self-evaluations of CHS staff. In Weifang, private CHS had more staff in both the young age group (<25) and the older group (>60) compared to staff at public CHS, probably because new private providers recruit recent graduates from medical school and retirees. There were no significant differences in education between public and private providers in these two cities. Different technical titles were found in City Y and Weifang, although the majority in both had primary or middle technical titles. The income gap between public and private employees in Weifang was less than in City Y: public employees in Y reported much higher income than private employees.

**Table 10:** Comparison of community health station staff in Weifang and City Y

**Indicators (%)**	**Weifang**				**City Y**			
	**All**	**Pub-C**	**Pri-C**	**Pri-Pub Difference**	**All**	**Pub-C**	**Pri-C**	**Pri-Pub Difference**
**Gender**								
Female	75.4	77.6	69.3	−8.29***	76.1	80.5	72.6	−7.93*
Male	24.6	22.4	30.7	8.29***	23.9	19.5	27.4	7.93
**Age**								
0-25	24.1	18.2	40.4	22.21***	24.3	22.2	26.1	3.92
26-39	50.2	53.0	42.5	−10.46***	36.6	38.9	34.8	−4.14
40-59	23.1	26.2	14.6	−11.54***	29.6	34.6	25.7	−8.94**
60+	1.2	0.4	3.5	3.11***	9.9	4.9	13.9	9.05***
**Working experience, total (years)**								
0-3	23.0	17.1	39.4	22.31***	24.3	21.6	26.5	4.90
4-9	17.7	16.8	20.2	3.39	16.4	10.3	21.3	11.03***
10-19	35.1	39.8	22.0	−17.87***	22.7	30.3	16.5	−13.75***
20+	22.5	24.7	16.7	−7.93***	35.9	36.8	35.2	−1.54
**Working years in any CHS**								
0-3	79.7	76.9	87.5	10.59**	65.3	58.4	70.9	12.49**
4-9	8.5	9.5	5.9	−3.56**	21.4	20.5	22.2	1.63
10-19	3.6	4.2	2.1	−2.08*	6.7	14.1	0.9	−13.18***
20+	0.6	0.4	1.0	0.67	3.6	4.3	3.0	−1.28
**Working years in the current CHS**								
0-3	60.5	53.6	79.4	25.84***	58.3	41.6	71.7	30.12***
4-9	10.8	10.1	12.5	2.43	14.5	14.1	14.8	0.73
10-19	14.7	18.6	4.2	−14.40***	14.9	29.2	3.5	−25.71***
20+	6.8	8.6	1.7	−6.85***	5.5	12.4	0.0	−12.43***
**Education**								
Middle health school	39.2	39.2	39.4	0.18	42.9	42.7	43.0	0.34
Junior college	46.0	44.8	49.5	4.72	38.1	36.8	39.1	2.37
Bachelor	14.5	15.3	12.2	−3.10	17.1	19.5	15.2	−4.24
Master and above	0.2	0.1	0.3	0.22	0.2	0.5	0.0	−0.54
**Technical Title**								
Primary	55.5	55.2	56.1	0.85	46.3	36.8	53.9	17.16***
Junior	29.0	31.0	23.7	−7.28**	33.7	45.4	24.3	−21.06***
Senior	4.6	4.8	4.2	−0.62	11.6	9.7	13.0	3.31
No	10.5	8.1	17.1	8.98***	7.2	7.6	7.0	−0.61
**Annual income (RMB)**								
<800	9.2	9.9	7.3	−2.54	7.2	13.5	2.2	−11.34***
800-1000	14.8	12.9	20.2	7.31***	13.3	8.6	17.0	8.31**
1000-1500	18.6	18.8	17.8	−1.07	21.2	11.9	28.7	16.80***
1500-2000	15.9	16.3	14.6	−1.67	18.3	17.3	19.1	1.83
2000-3000	8.1	9.0	5.6	−3.40*	10.4	15.7	6.1	−9.59***
>3000	31.7	31.0	33.8	2.82	27.5	30.3	25.2	−5.05
**Satisfaction with current job**								
Very satisfied	17.4	14.7	25.1	10.42***	16.4	13.5	18.7	5.18
Satisfied	45.2	43.1	50.9	7.76**	57.1	43.2	68.3	25.02***
Dissatisfied	34.5	38.6	23.3	−15.21***	22.7	37.8	10.4	−27.40***
Very dissatisfied	2.6	3.2	1.0	−2.12*	3.1	5.9	0.9	−5.08***
**CHS staffs’ average self-assessed degree of knowledge about residents**								
Knowing residents in the served area#	69.4	66.8	76.6	9.81***	71.4	65.8	76.0	10.23***
Willingness of residents to choose this CHS as the first visit#	64.0	62.0	69.5	7.51***	55.8	50.4	60.4	10.02***

Notes: 1) ***, **, * refer to 1%, 5% and 10% statistical significance level, respectively; 2) #here we report the averages of CHC staffs’ self assessed degree of knowing residents in the served area and willingness of residents to choose this CHS as the first visit and relevant mean test (‘ttest’ in Stata) results (P-values) for public-private differences, since staff answered with a continuous response (between 1-100%) for these two questions; 3) the summations for each category may not equal 100% because of missing values.

Private employees, however, reported higher job satisfaction than public employees. This may be because public employees tend to compare themselves with (former and future) colleagues in public hospitals who tend to enjoy better opportunities for career development than employees at CHS. Among private providers reporting dissatisfaction, the top-cited reasons were lack of social security, limited career paths, low income, and feeling overworked. Compared to staff at public CHS, private providers showed more confidence that they understood the local community and that their patients would re-visit them. 64 percent of Weifang staff but only 56 percent of City Y staff believed that residents would choose the CHS for first-contact care, with less of an ownership difference in Weifang.

Table 11 presents the results of multivariate regression models examining the factors associated with CHS staff job satisfaction and CHS performance scores. Job satisfaction is higher at private CHS, with less of a public-private difference in Weifang than in City Y. Other factors associated with job satisfaction include the specific job of the staff member (e.g. technician vs. general practitioner), age, and experience. The final column shows that work experience at the current CHS is positively associated with a better CHS performance score. Private CHS in City Y score significantly lower than other CHS, but this is not true for Weifang, which is consistent with the results reported earlier.

**Table 11:** Factors associated with staff satisfaction and performance scores of community health stations in Weifang and City Y

**Variables**		**Dependent variable: Probability satisfied with current job**	**Dependent variable: CHS performance score**
		**Logit**	**OLS**
Weifang		0.153	6.836
		(0397)	(1.787)***
Private		1.499	−5.539
		(0.418)***	(2.946)*
Private*Weifang		−0.855	4.375
		(0.598)	(3.258)
Male		0.008	−0.388
		(0.201)	(0.991)
Age		0.019	−0.022
		(0.009)**	(0.159)
Age squared			0.000
			(0.002)
Working years at current CHS		−0.024	0.156
		(0.019)	(0.083)*
Dummy for junior college		0.097	0.543
		(0.218)	(0.703)
Dummy for college and above		−0.049	−1.964
		(0.294)	(1.783)
Dummy for job positions	manager	−0.024	0.741
		(0.314)	(1.015)
	logistics worker	−1.059	0.437
		(0.282)***	(0.952)
	general practitioner	−0.660	−0.382
		(0.270)**	(1.114)
	technician	−0.247	3.595
		(0.465)	(1.531)**
	nurse	−0.386	−0.584
		(0.277)	(0.755)
	pharmacist	−0.129	−0.123
		(0. 319)	(0.873)
	other general	−0.661	−0.472
		(0.266)**	(1.047)
Constant		0.386	76.542
		(0.492)	(3.251)***
Observations		1298	661
R-squared			0.26

Notes: 1) Standard errors in parentheses are clustered at the CHS level; 2) ***, **, * refer to 1%, 5% and 10% statistical significance level, respectively.

## Discussion

Contracting with private providers is increasingly discussed as an option for efficiently closing gaps in primary care coverage in low- and middle- income countries [29]. Private sector participation and management practices, in the health sector and beyond, can be attractive when government agencies and providers appear to be over-extended and/or inefficient [30]. Many scholars have argued that given appropriate financial incentives, the private sector will supply appropriate services to patients. For example, Meng, Liu, and Shi [8] found that in 1997, Chinese village clinics subsidized 0.46 RMB per resident were willing to provide the defined package of preventive services. Some observers have argued that private providers may even be willing to supply certain preventive services without payment in order to enhance their reputation and expand market share [31].

Our study addresses these important policy questions through a case study of contracting for preventive health services in urban China. We find, for example, that it is important to take into account the socioeconomic characteristics of the community served by providers of different ownership forms. In Weifang, residents in the communities served by private CHS are more likely to be uninsured and to report poor health, compared to residents in communities served by a government-owned CHS. Comparisons also need to take account of differences in provider size when discussing performance. In Weifang, private CHS tend to be smaller than public CHS and to have somewhat lower performance scores; but when comparing public and private CHS of similar size, they have performance scores so close as to be statistically indistinguishable.

In our study, the same basic purchasing mechanism with the same contract price – 10 RMB per resident – was associated with different performance in Weifang and City Y. In Weifang, where private providers were on a more equal footing with public providers, there were essentially no ownership differences in contracted performance. In contrast, the private sector in City Y did not perform as well.

A key concern in China is that providers are overly focused on curative care, where they can earn fee-for-service revenue, and tend to neglect preventive health services and population health outreach services, which may not generate any revenue. City Y policymakers complained that private CHS preferred to focus on profitable clinical treatment and were reluctant to provide preventive services, presumably because they were unsure that payment would compensate for the direct and opportunity costs in terms of foregone clinical service revenue. The Weifang experience shows that the purchasing agency might be able to overcome this barrier by providing technical and financial support, as well as calling for residents’ participation and community involvement, which can be important in helping private providers earn the trust of neighborhood residents.

Since in Weifang both public and private CHS provided preventive and outreach services when paid to do so, our case study is consistent with the idea that both public and private CHS could be willing to provide preventive services if the expected marginal revenue matches or exceeds that to be expected from clinical services, and the provider has clear guidelines and support. Our case study, particularly the synthesis of interviews with policymakers and providers, suggest that the provider contract should clearly state objectives and performance criteria. Items which can be quantified and for which quality can be measured objectively should be given priority, because it is easier to pay for what both the purchaser and seller can easily measure. Improved provider payment methods should also be considered, in part to minimize the unintended consequences under fee-for-service for services not explicitly rewarded in the performance contract.

We find that residents do not generally trust the quality of CHS, although residents who had visited a CHS showed more positive attitudes. These results are consistent with previous literature. For example, Yang and Yang [32] studied community health organizations in five cities, finding that that urban Chinese “do not generally trust community health service centers” [32], p.624] and that patients who were poor, unemployed, or uninsured were most likely to use community health centers. In our Shandong data, residents were more willing to do a routine health exam at community health stations if they were of low socioeconomic status (as measured either by education or income) and if the station was privately owned. Residents who had visited a CHS showed more positive attitudes toward private providers than public providers.

There are several limitations of this study. The results are based on cross-sectional data. Without systematic historical data or a dedicated baseline survey in both intervention and control areas, we could not conduct a more comprehensive comparison with a difference-in-difference methodology. Because of the difficulty with objective quality assessment, some conclusions are tentative. Further research should examine the impact of non-contracted dimensions of service and quality.^j^

## Conclusions

Case studies such as the one described in this paper provide evidence regarding the complicated challenge of effective public stewardship of private providers, which advocates claim can improve performance and lead private providers to serve policy goals, if incentives are designed appropriately [33]. In some areas where government healthcare resources are insufficient, contracting out may allow expansion toward universal coverage more rapidly and with lower government investment.^k^ To assess these claims in any specific institutional context, data should be collected at baseline and during implementation to allow scientific evaluation of the contracting outcomes, and the contracting arrangements themselves should be simple, clear, and enforceable.

The next phase of China’s health reforms calls for more active engagement with the private sector. Success in this endeavor calls for enhanced information, transparency, monitoring, training, communication, incentives, and ownership-neutral access to career development and social security. In health systems such as China’s—which has been long dominated by government providers and a legacy of suspicion of private enterprise—social marketing, public policy support, and accreditation or other guarantees of qualified service can help residents eliminate discriminatory perceptions of private providers. A major concern among private providers in urban China is competition for human resources, technology, and market access (i.e., to be included in the provider network for social insurance). Addressing these concerns could be an important step toward harnessing the entrepreneurial spirit of private providers in pursuit of better primary care, while rigorous evaluations help to defend the public interest from profit-seeking at the expense of patient welfare.

## Endnotes

^a^ Community health centers are larger than community health stations in terms of beds, personnel, equipment, and responsibility for population health services. In some cases, a community health center owns and manages the associated health stations; in other cases, the stations are legally and functionally independent. To simplify exposition, we use the abbreviation CHS for both community health stations and community health centers.

^b^ This policy represents the first time that private providers received public funds for capital investments in Weifang.

^c^ To assure objectivity and provide a quality check on the evaluation process, 30 percent of CHS were re-visited by a separate provincial-level evaluation team. The final scores are S=S_1_/S_2_*S_2_, where S1 represents the provincial team score and S2 the local evaluation for each CHS.

^d^ Only public hospitals were permitted to run community health centers.

^e^ The Ministry of Health set 150 m^2^ as the minimum required clinic space for a CHS, and listed the equipment that a CHS was required to possess.

^f^ Unfortunately we were not able to obtain data that allowed direct comparison of spending for the same patient diagnosis at public and private CHS. Because of the EML policy in Weifang, we do know that patient out-of-pocket payments were likely to be lower in Weifang than in City Y.

^g^ In Beijing, where CHS development started a couple years before that in Weifang, 78.4% of interviewed patients discharged from hospitals had heard of CHS and 35.5% of them had visited CHS (Ding et al. 2009).

^h^ Interestingly, separately examining the subset of patients in Pub-C and Pri-C who had previously visited the CHS and thus may have some knowledge and medical needs, we find different associations between insurance and willingness to visit the CHS: in Pub-C, the uninsured are less likely to do routine exams at the CHS; in Priv-C, the uninsured are more likely to do a routine exam at the CHS. This may reflect unmeasured differences in the resident population as well as differences in the CHS by ownership form.

^i^ Fewer than one in ten residents knew CHS were open until 8 pm. That high proportions of respondents reported willingness or desire to change community health care providers also shows the gulf of trust between patients and grassroots providers in China.

^j^ For example, anecdotal evidence suggests private providers may be more likely than public providers to over-prescribe antibiotics and intravenous injections.

^k^ In Weifang, contracting out to private providers cost the government about one-tenth of what it would have cost to build new government clinics from scratch for the communities served by private CHS.

## Abbreviations

CHS: Community Health Station (or Community Health Center); City Y: Comparison city for Weifang; EML: Essential Medication List; GDP: Gross Domestic Product; RMB: Ren Min Bi Yuan; Pub-C: Public (Government-owned) Community Health Station; Pvt-C: Private Community Health Station.

## Competing interests

The authors declare that they have no competing interests.

## Authors’ contributions

YW and KE conceived of the study and drafted the manuscript. ZY participated in the study design, helped YW collect the survey and provider data, and contributed to revisions of the manuscript. QZ performed the statistical analysis and contributed to revisions of the manuscript. All authors read and approved the final manuscript.

## Supplementary Material

Additional file 1: Appendix Table S1. Descriptive statistics for CHS in Weifang and Y in 2009. **Table S2.** F-tests for regressions in Table 5. (DOC 69 kb) Click here for file
