# Supplementary material for: Contracting with private providers for primary care services: evidence from urban China
Source: Health Econ Rev. 2013 Jan 17;3:1. doi: 10.1186/2191-1991-3-1 (PMC3599686; doi:10.1186/2191-1991-3-1)
Supplement: Additional file 1: Appendix Table S1. — Descriptive statistics for CHS in Weifang and Y in 2009. Table S2. F-tests for regressions in Table 5. (DOC 69 kb) [file 2191-1991-3-1-S1.doc]

**Appendix Table S1. Descriptive statistics for CHS in Weifang and Y in 2009**

|  | Weifang | | City Y | |
| --- | --- | --- | --- | --- |
|  | government | private | government | private |
| CHS performance scores | 81.97 | 82.28 | 76.07 | 69.68 |
|  | (4.84) | (5.28) | (6.47) | (17.45) |
| Percentage of fiscal subsidy | 0.45 | 0.46 | 0.09 | 0.10 |
|  | (0.27) | (0.17) | (0.08) | (0.11) |
| Separating prescribing from dispensing (=1 if yes) | 0.35 | 0.27 | 0.00 | 0.21 |
|  | (0.49) | (0.45) | 0.00 | (0.41) |
| Designated for medical insurance (=1 if yes) | 0.72 | 0.78 | 0.77 | 0.53 |
|  | (0.45) | (0.42) | (0.43) | (0.50) |
| Percentage of income from selling drugs | 0.35 | 0.22 | 0.39 | 0.53 |
|  | (0.31) | (0.19) | (0.27) | (0.30) |
| Number of staffs | 9.93 | 9.67 | 11.18 | 8.14 |
|  | (6.99) | (4.13) | (6.74) | (2.92) |
| Housing | 512.56 | 305.68 | 313.29 | 204.29 |
|  | (914.90) | (292.14) | (259.22) | (107.30) |
| Fixed asset | 141.46 | 45.54 | 31.28 | 22.95 |
|  | (327.47) | (61.62) | (41.44) | (57.69) |
| Number of beds | 6.50 | 6.96 | 7.36 | 7.07 |
|  | (6.17) | (2.19) | (4.10) | (3.74) |
| Return to fixed assets (Revenue/Fixed asset) | 1.65 | 2.42 | 14.36 | 3.92 |
|  | (2.24) | (2.86) | (20.30) | (4.32) |
| Profit rate of fixed assets (Profit/Fixed asset) | 0.12 | 0.86 | 11.28 | 0.52 |
|  | (2.01) | (1.77) | (21.12) | (1.43) |
| Observations | 29 | 27 | 22 | 43 |

Notes: Standard deviations in parentheses.

**Appendix Table S2. F-tests for regressions in Table** 5

| Joint test for | Private | Weifang | Private*Weifang | P-value |
| --- | --- | --- | --- | --- |
| (1) | √ | √ | √ | 0.0001 |
|  |  | √ | √ | 0.0000 |
|  | √ |  | √ | 0.1704 |
| (2) | √ | √ | √ | 0.0000 |
|  |  | √ | √ | 0.0000 |
|  | √ |  | √ | 0.1450 |
| (3) | √ | √ | √ | 0.0000 |
|  |  | √ | √ | 0.0000 |
|  | √ |  | √ | 0.1344 |
| (4) | √ | √ | √ | 0.0001 |
|  |  | √ | √ | 0.0000 |
|  | √ |  | √ | 0.2203 |
| (5) | √ | √ | √ | 0.0001 |
|  |  | √ | √ | 0.0000 |
|  | √ |  | √ | 0.2853 |
